# Tai Chi for coronavirus disease 2019 in recovery period: A protocol for systematic review and meta analysis

**DOI:** 10.1097/MD.0000000000021459

**Published:** 2020-08-07

**Authors:** Yu Shi, Dengpeng Wen, Hui Wang, Puyue Zhang, Yanmei Zhong, Donghao Liu, Deqi Zhou

**Affiliations:** a College of Acupuncture and Moxibustion and Tuina, Chengdu University of Traditional Chinese Medicine; b Beibei District Traditional Chinese Medicine Hospital of Chongqing; c School of Medical Information Engineering, Chengdu University of Traditional Chinese Medicine; d School of Basic Medical Science, Chengdu University of Traditional Chinese Medicine.

**Keywords:** coronavirus disease 2019, systematic review, tai chi

## Abstract

**Background:**

Assessing the effectiveness and safety of Tai Chi for coronavirus disease 2019 (COVID-19) in recovery period is the main purpose of this systematic review protocol.

**Methods:**

The following electronic databases will be searched from inception to April 2020: MEDLINE, Ovid, EMBASE, the Cochrane Library, the Allied and Complementary Medicine Database, Chinese National Knowledge Infrastructure, Chinese Biomedical Literature Database, VIP Database and Wanfang Database. In addition, Clinical trial registries, like the Chinese Clinical Trial Registry, the Netherlands National Trial Register and ClinicalTrials.gov, will be searched for ongoing trials with unpublished data. No language restrictions will be applied. The primary outcome will be the time of disappearance of main symptoms (including fever, asthenia, cough disappearance rate, and temperature recovery time), and serum cytokine levels. The secondary outcome will be the accompanying symptoms (such as myalgia, expectoration, stuffiness, runny nose, pharyngalgia, anhelation, chest distress, dyspnea, crackles, headache, nausea, vomiting, anorexia, diarrhea) disappear rate, negative COVID-19 results rate on 2 consecutive occasions (not on the same day), CT image improvement, average hospitalization time, occurrence rate of common type to severe form, clinical cure rate, and mortality. Two independent reviewers will conduct the study selection, data extraction and assessment. Review manager software V.5.3 will be used for the assessment of risk of bias and data synthesis.

**Results:**

The results will provide a high-quality synthesis of current evidence for researchers in this subject area.

**Conclusion:**

The conclusion of the study will provide an evidence to judge whether Tai Chi is effective and safe for COVID-19 in recovery period.

**Ethics and dissemination:**

This protocol will not evaluate individual patient information or infringe patient rights and therefore does not require ethical approval. Results from this review will be disseminated through peer-reviewed journals and conference reports.

PROSPERO registration number CRD42020181456.

## 1 Introduction

Coronavirus disease 2019(COVID-19) is a virus (more specifically, a coronavirus) identified as the cause of an outbreak of respiratory illness first detected in Wuhan, China. The new type of pneumonia is spreading fast and by February 7, 2020, 43103 cases of COVID-19 have been confirmed in 25 countries [World Health Organization. Novel coronavirus (2019-nCoV). Situation report 22. Geneva, Switzerland: World Health Organization, 2020.]. The mean incubation period of COVID-19 is 5.2 days.^[1]^ It usually begins with nonspecific symptoms like fever, dry cough and fatigue, and multiple systems like respiratory, gastrointestinal and musculoskeletal may be involved.^[2]^ The diagnostic criteria are as follows:

(1) epidemiologic history (travel and/or residence history in Wuhan or exposure history to patients with fever and respiratory symptoms from Wuhan within 14 days before the onset of illness);
(2) clinical manifestations (fever, imaging characteristics of pneumonia, and/or normal or decreased white blood cell count or decreased lymphocyte count); and
(3) laboratory diagnosis (real-time polymerase chain reaction revealed positive detection of COVID-19 in throat swabs or lower respiratory tract).

There is no validated treatment for COVID-19 currently. The main methods are keeping vital signs, maintaining oxygen saturation and so on, which are symptomatic and supportive care. Meanwhile, some complications such as secondary infections and organs failure need to be taken into consideration. With these strategies, the patient will gradually recover from the disease.

Tai Chi is a form of traditional Chinese low-to-moderate-intensity mind-body exercise with a long practicing history for body and mind fitness in the East.^[3]^ Many studies have shown that Taichi is effective in treating chronic conditions, like immunity and infection,^[4]^ cardiovascular conditions^[5]^ and chronic musculoskeletal pain conditions.^[6]^

This review aims to systematically evaluate the effectiveness and safety of Tai Chi for COVID-19 in recovery period by including multiple clinical trials published over the past 10 years.

## 2 Methods and analysis

### 2.1 Study registration

This systematic review protocol has been registered with PROSPERO 2020 (registration number: CRD42020187422). And the protocol report is in the base of the Preferred Reporting Items for Systematic Reviews and Meta-Analyses (PRISMA) Protocols declaration guidelines.^[7]^ The review will be performed in line with the PRISMA declaration guidelines.^[8]^

### 2.2 Inclusion criteria for study selection

#### 2.2.1 Type of study

All randomised controlled trials (RCTs) about Tai Chi for COVID-19 in recovery period will be included. There is no language limitation. Non-RCTs, quasi-RCTs, case series, reviews, animal studies and any study with a sample size of less than ten participants will be excluded.

#### 2.2.2 Type of participant

Patients with COVID-19 in recovery period, regardless of sex, age, race or educational and economic status, will be included in the review.

#### 2.2.3 Type of interventions

Experimental intervention will include Tai Chi therapy. Control interventions will be western medicine therapy.

#### 2.2.4 Type of outcome measures

The primary outcome will be the time of disappearance of main symptoms (including fever, asthenia, cough disappearance rate, and temperature recovery time), and serum cytokine levels. The secondary outcome will be the accompanying symptoms (such as myalgia, expectoration, stuffiness, runny nose, pharyngalgia, anhelation, chest distress, dyspnea, crackles, headache, nausea, vomiting, anorexia, diarrhea) disappear rate, negative COVID-19 results rate on 2 consecutive occasions (not on the same day), CT image improvement, average hospitalization time, occurrence rate of common type to severe form, clinical cure rate, and mortality.

### 2.3 Search methods for identification of studies

#### 2.3.1 Electronic data sources

The following electronic databases will be searched from inception to April 2020: MEDLINE, Ovid, EMBASE, the Cochrane Library, the Allied and Complementary Medicine Database (AMED), Chinese National Knowledge Infrastructure (CNKI), Chinese Biomedical Literature Database (CBM), VIP Database and Wanfang Database. In addition, Clinical trial registries, like the Chinese Clinical Trial Registry (ChiCTR), the Netherlands National Trial Register (NTR) and ClinicalTrials.gov, will be searched for ongoing trials with unpublished data. No language restrictions will be applied.

#### 2.3.2 Searching other resources

A reference list of potential, qualified studies and related system reviews will be manually retrieved and reviewed. We will contact the author for the up-to-date data for the ongoing RCTs. Furthermore, relevant conference proceedings will be evaluated to identify studies related to this review.

#### 2.3.3 Search strategy

The search strategy for PubMed is shown in Table 1. The following search keywords will be used: Tai Chi (eg, “Tai-ji” or “Chi, Tai” or “Tai Ji Quan” or “Ji Quan, Tai” or “Quan, Tai Ji” or “Taiji” or “Taijiquan” or “T’ai Chi”or “Tai Chi Chuan”); COVID-19 (eg, “2019-nCoV” or “Wuhan coronavirus” or “SARS-CoV-2” or “2019 novel coronavirus” or “COVID-19 virus” or “coronavirus disease 2019 virus” or “COVID19 virus” or “Wuhan seafood market pneumonia virus”); randomized controlled trial (eg, “randomized controlled trial” or “controlled clinical trial” or “random allocation” or “randomized” or “randomly” or “double-blind method” or “single-blind method” or “clinical trial”.

**Table 1:**
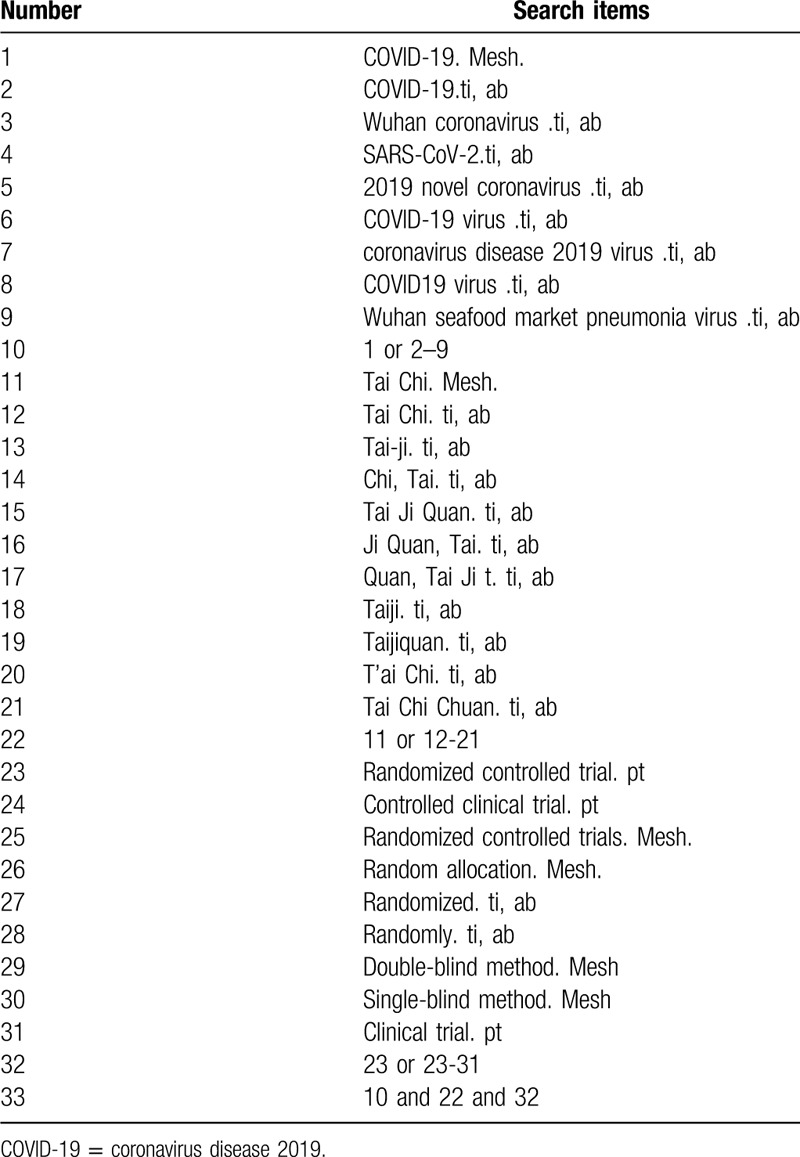
Search strategy for the PubMed database.

### 2.4 Data collection and analysis

#### 2.4.1 Selection of studies

**Figure 1:**
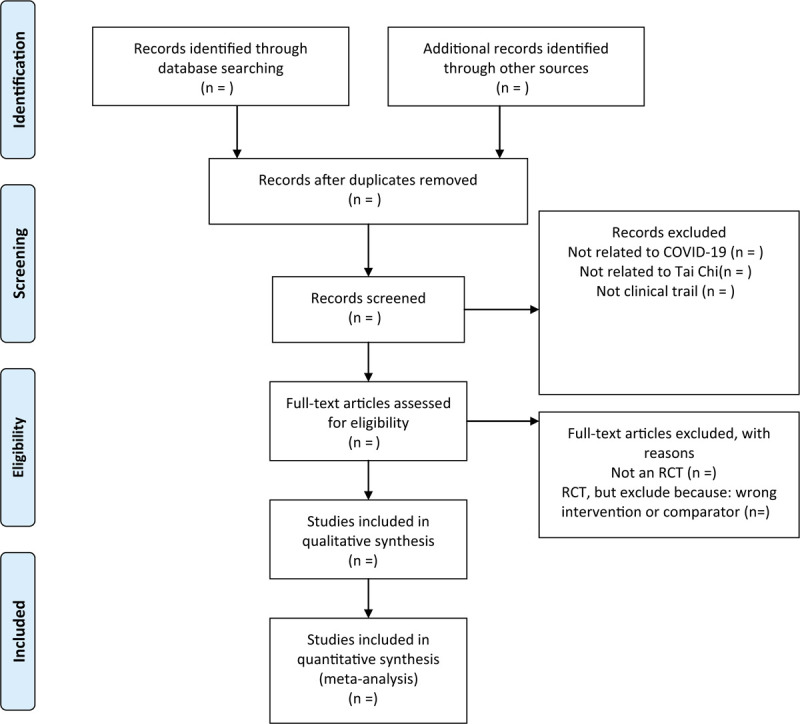
The PRISMA-P flow chart of the study selection process. PRISMA-P = the Preferred Reporting Items for Systematic Reviews and Meta-Analyses Protocols.

Two trained reviewers will review and screen the titles and abstracts of all searched studies independently. The duplicate records and ineligible studies will be eliminated to determine whether they meet the predefined inclusion criteria. A PRISMA flow diagram will be used to show the study selection process in Figure 1.

#### 2.4.2 Data extraction and management

Two independent authors will extract data and fill in the data extraction form. These data will be obtained: general information, participants, methods, interventions, outcomes, results, adverse events, conflicts of interest, ethical approval and other information. If the data is insufficient, the authors will be contacted. Any disagreements will be resolved by discussion between the 2 authors, and further disagreements will be arbitrated by the third author.

#### 2.4.3 Assessment of risk of bias and reporting of study quality

Two reviewers will independently access the quality of included literature and complete the Standards for Reporting Interventions in Clinical Trials of Acupuncture checklist with the Cochrane collaboration risk-of-bias assessment method.^[9]^

#### 2.4.4 Measures of treatment effect

Review manager software (RevMan) V.5.3 will be used for data analysis and quantitative data synthesis. For continuous data, the mean difference (MD) or standard MD (SMD) will be used to measure the treatment effect with 95% CIs, if no heterogeneity is found. The random-effects model will be used if there is significant heterogeneity. A risk ratio (RR) with 95% CIs for analysis will be used for dichotomous data.

#### 2.4.5 Unit of analysis issues

The units of each outcome from different trials will be converted to the International System of Units before statistical analysis.

#### 2.4.6 Management of missing data

The authors will be contacted for the missing part. This will be documented and the available data will be extracted and analyzed if the missing data cannot be obtained.

#### 2.4.7 Assessment of heterogeneity

*I*^2^statistic will be used to quantify inconsistencies among the included studies. If the *I*^2^ value is less than 50%, it indicates that the studies have no significant statistical heterogeneity. And if the *I*^2^ value exceeds 50%, the studies are considered to have significant statistical heterogeneity among the trial and no meta-analysis need to be performed. We will conduct subgroup analysis to explore possible causes.

### 2.5 Assessment of reporting bias

Funnel plots will be used to access the reporting biases if there are over 10 trials included in the meta-analysis.^[10]^

### 2.6 Data synthesis

RevMan V.53 will be used for data synthesis. The random-effects model will be used if the *I*^2^ value is no less than 50%. The fixed-effects model will be used if the heterogeneity tests show little statistical heterogeneity. If there is meaningful heterogeneity that cannot be explained by any assessment, meta-analysis will not be performed.

### 2.7 Subgroup analysis

There is no pre-subgroup plan. Subgroup analysis will be conducted if data are available. Factors such as different types of control interventions and different outcomes will be considered.

### 2.8 Sensitivity analysis

Sensitivity analysis will be conducted to test the robustness of the review conclusions if possible. We will evaluate the impacts of sample size, study design, methodological quality, and missing data.

### 2.9 Grading of evidence quality

The Grading of Recommendations Assessment approach will be used to judge the quality of the evidence for all outcomes.^[11]^ Risk of bias, heterogeneity, indirectness, imprecision and publication bias will be assessed. The assessments will be classified into 4 levels: high, moderate, low or very low.

### 2.10 Ethics and dissemination

This protocol will not evaluate individual patient information or infringe patient rights and therefore does not require ethical approval. Results from this review will be disseminated through peer-reviewed journals and conference reports.

## 3 Discussion

This systematic review will be the first to assess the effectiveness and safety of Tai Chi for COVID-19 in recovery period. The review contains 4 sections: identification, study inclusion, data extraction, and data synthesis. This review will aid doctors in the decision-making process for treating patients with COVID-19 in recovery period, and will provide information for patients and health policy makers.

## Acknowledgments

None declared.

## Author contributions

YS, DPW, and HW have made contributions to this manuscript and are the joint first authors. Funding has been obained by DQZ. YS, DPW, and HW have drafted the protocol. PYZ, YMZ, and DHL have made the search strategy and it will be conducted by them. DPW and HW will obtain copies of the studies. The studies will be screened by PYZ and DHL. YS and HW will perform the data extraction from the studies. And YMZ and DHL will conduct the data into RevMan. Analyses will be done by YS. YS, DPW and HW will draft the final review. DQZ will act as an arbiter in the study selection stage. All authors have read and approved the final manuscript.
